# Comparative analysis for renal stereotactic body radiotherapy using Cyberknife, VMAT and proton therapy based treatment planning

**DOI:** 10.1002/acm2.12308

**Published:** 2018-03-14

**Authors:** Atallah Baydoun, Neha Vapiwala, Lee E. Ponsky, Musaddiq Awan, Ali Kassaee, David Sutton, Tarun K. Podder, Yuxia Zhang, Donald Dobbins, Raymond F. Muzic, Bryan Traughber, Mitchell Machtay, Rodney Ellis

**Affiliations:** ^1^ Department of Internal Medicine Case Western Reserve University School of Medicine Cleveland OH USA; ^2^ Department of Internal Medicine Louis Stokes VA Medical Center Cleveland OH USA; ^3^ Department of Biomedical Engineering Case Western Reserve University Cleveland OH USA; ^4^ Abramson Cancer Center University of Pennsylvania Philadelphia PA USA; ^5^ Department of Radiation Oncology University of Pennsylvania Philadelphia PA USA; ^6^ Department of Radiation Oncology Case Western Reserve University School of Medicine Cleveland OH USA; ^7^ Department of Urology Case Western Reserve University School of Medicine Cleveland OH USA; ^8^ University Hospitals Seidman Cancer Center Case Comprehensive Cancer Center OH USA; ^9^ Case Center for Imaging Research University Hospitals Case Medical Center Cleveland OH USA; ^10^ Department of Radiology Case Western Reserve University School of Medicine Cleveland OH USA

**Keywords:** proton therapy, renal cell carcinoma, SBRT, VMAT

## Abstract

**Purpose:**

We conducted this dosimetric analysis to evaluate the feasibility of a multi‐center stereotactic body radiation therapy (SBRT) trial for renal cell carcinoma (RCC) using different SBRT platforms.

**Materials/methods:**

The computed tomography (CT) simulation images of 10 patients with unilateral RCC previously treated on a Phase 1 trial at Institution 1 were anonymized and shared with Institution 2 after IRB approval. Treatment planning was generated through five different platforms aiming a total dose of 48 Gy in three fractions. These platforms included: Cyberknife and volumetric modulated arc therapy (VMAT) at institution 1, and Cyberknife, VMAT, and pencil beam scanning (PBS) Proton Therapy at institution 2. Dose constraints were based on the Phase 1 approved trial.

**Results:**

Compared to Cyberknife, VMAT and PBS plans provided overall an equivalent or superior coverage to the target volume, while limiting dose to the remaining kidney, contralateral kidney, liver, spinal cord, and bowel.

**Conclusion:**

This dosimetric study supports the feasibility of a multi‐center trial for renal SBRT using PBS, VMAT and Cyberknife.

## 1 INTRODUCTION

With an incidence of 62 700 new cases in 2016, kidney and renal pelvic cancers account for around 4% of newly diagnosed cancers in the USA.^1^ Renal cell carcinoma (RCC) is the predominant and most lethal histology accounting for about 87% of these malignancies.^2^, ^3^ Historically, RCC has been labeled as a “radio‐resistant tumor” and surgical nephrectomy was considered the cornerstone of treatment for RCC. A gradual shift in RCC treatment modalities began in the 1990s with the introduction of laparoscopic nephrectomy, high intensity focused ultrasound, cryoablation, radiofrequency ablation, and tyrosine kinase inhibitors but radiation remained rarely used.^2^

Two main factors contributed to the underutilization of radiotherapy in treating RCCs: the high metastatic potential of the cancer and an inability to safely deliver high dose curative intent radiation to the primary tumor due to the anatomic proximity of the kidneys to other radio‐sensitive structures such as small bowel. However, the successful use of both image‐guided conventional radiotherapy and more recently stereotactic radio‐surgery in the local treatment of extracranial and intracranial RCC metastases, respectively, challenged the role of radiation therapy in the management of RCC. While previous literature suggested that RCC is radio‐resistant to small fraction sizes,^4^ higher fraction dose delivered through Stereotactic Body Radiation Therapy (SBRT) can achieve promising rates of local control and acceptable toxicity.^5^

SBRT allows for the accurate delivery of high dose radiation to specific extracranial targets while potentially avoiding toxic doses to adjacent structures. The use of SBRT in the treatment of local RCC was first reported by Qian et al.^5^ who achieved a local control rate of 93% at a mean follow‐up of 12 months.^6^ A few years later, we reported our experience of a Phase I trial of SBRT using the Cyberknife platform and emphasizing the safety and efficacy in non‐surgical RCC treatment.^7^ SBRT can also be delivered with other platforms including volumetric modulated arc therapy (VMAT) or pencil beam scanning (PBS) proton therapy with each field covering the target uniformly. Dosimetric differences between these platforms have not been well studied in RCC and remain a major barrier for the implementation of large multi‐institutional trials. Therefore, we conducted this study to assess the dosimetric feasibility of using non‐robotic platforms for delivering curative‐intent renal SBRT as a precursor for a future multi‐institutional trial.

## 2 MATERIALS AND METHODS

### 2.A Patients selection

An institutional review board‐approved phase I dose‐escalation trial of SBRT using Cyberknife for primary treatment of non‐surgical patients with localized RCC (NCT00458484) was initiated at our facility (Institution 1) since June 2006. The primary tumor was deemed to be resectable by an experienced urologic oncologist, but patients were referred to this phase I trial due to underlying medical conditions prohibiting surgical excision such as low probability of tolerating the general anesthesia, the surgery itself, or the postoperative recovery period.^7^ At the time of the diagnostic biopsies, at least three fiducials markers were placed within and around the renal mass.^7^ Within 1 week after fiducials insertion, computerized tomography (CT) simulation was acquired. Patients were treated to the primary tumor plus 0–3 mm margins with radiotherapy doses of 24, 32, 40, and 48 Gy in four fractions. Inclusion and exclusion criteria, radiation technique, dosimetric planning, and initial results were previously reported.^7^ The institutional review board also approved the current dosimetric study. Among 19 patients with unilateral RCC treated according to the phase I trial with 48 Gy in four fractions, ten patients were randomly selected for this study. CT simulation images were then anonymized and shared with Institution 2.

### 2.B Treatment planning and dosimetric variables

**Figure 1:**
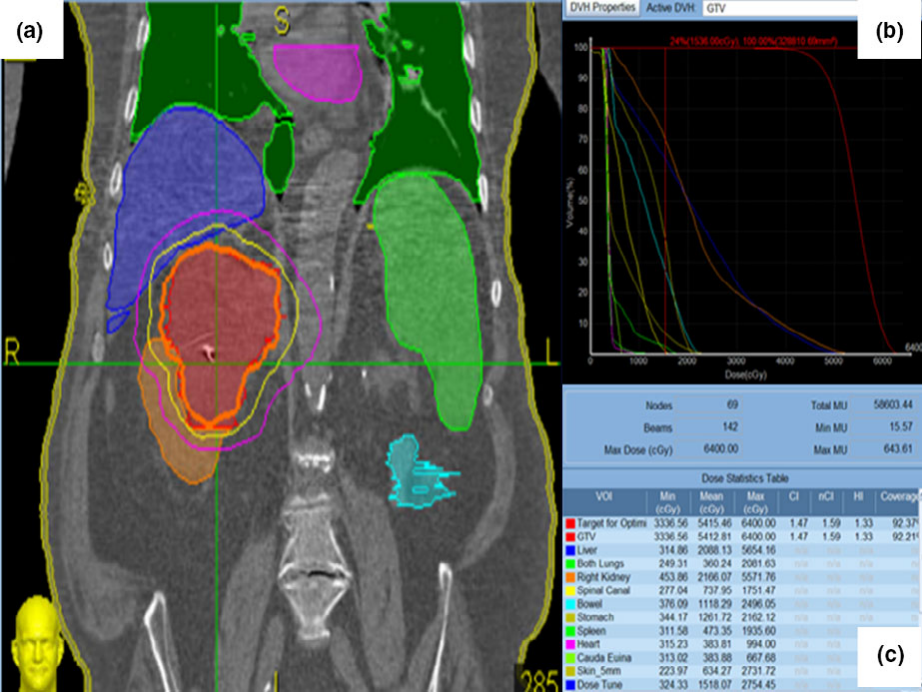
Planning treatment (a) with the corresponding dose‐volume histogram (b and c) in an 81 year‐old male patient with 9.5 cm right posterior upper pole Renal cell carcinoma, Clear cell type, Furman Grade 2 deemed medically inoperable. Orange, yellow and magenta lines in (a) corresponds respectively to 48, 36 and 24 Gy isodose line.

Using the anonymized CT images, treatment planning was performed using five different platforms with a prescription dose to the planning target volume (PTV) of 48 Gy in three fractions. These platforms included: Cyberknife (Accuray Inc., Sunnyvale, CA) and VMAT (Elekta Medical Stockholm, Sweden) at Institution 1, and Cyberknife, VMAT (Varian Medical Systems, Inc. Palo Alto, CA), and single‐field uniform dose PBS proton therapy (Ion Beam Applications, Louvain‐la‐Neuve, Belgium) at Institution 2. For planning purposes, VMAT used two arcs of 180 degrees each along with mirrored collimators. For PBS, two perpendicular beam angles were used. Fiducials would theoretically be available for PBS daily imaging and a 3.5% + 1 mm uncertainty range along the beam line directions was used. For all other modalities, the clinical target volume (CTV) to PTV expansion was 0–3 mm at the discretion of the planning radiation oncologist. A single PTV expansion for each patient was used at each institution for Cyberknife and VMAT. In addition to delineating the planning target volume (PTV) on each affected kidney, we also delineated V_normal_ defined as the total volume of the affected kidney minus the PTV. The organ at risk (OAR) dose constraints used for treatment planning in both institutions were similar to those published in the Phase I trial^7^ and are summarized in Table 1. For each platform, we extracted the percentage of the PTV receiving 100% of the prescribed dose [V_100%_(%)] and the percentage of V_normal_ receiving a dose of 14 Gy [V_14Gy_(%)]. In both institutions, priority was given at the planning level for PTV coverage over OAR sparing. In addition, we calculated the maximum dose delivered to 1 cc of the bowel (D_1cc_‐Bowel), 1 cc of the stomach (D_1cc_‐Stomach), 0.3 cc of the spinal cord (D_0.3cc_‐Cord), and five percent of the volume of the contralateral kidney (D_5%_‐Contralateral K.). Finally, for the liver, we calculated the percentage of liver volume receiving a total dose of 17 Gy or higher [V_17Gy_‐Liver (%)]. Sample contours are shown in Fig. 1.

**Table 1:** Dose specifications for normal organs

Organ	Dose specification
Bowel	≤1 cc can receive 2400 cGy
Cord	**≤**0.3 cc can receive 2000 cGy
Stomach	**≤**1.0 cc can receive 2200 cGy
Liver	**≤**66.67% of the total liver volume can receive 1700 cGy
Contralateral Kidney	**≤**5% of the total contralateral kidney can receive 1400 cGy

### 2.C Statistical analysis

For each of the seven variables defined above (V_100%_, V_14Gy_, D_1cc_‐Bowel, D_1cc_‐Stomach, D_0.3cc_‐Cord, D_5%_‐Contralateral K., V_17Gy_‐Liver) we calculated the mean and the standard deviation (SD) across all 10 patients for each treatment planning platform. For statistical testing, V_100%_ across different platforms was considered paired as the planning measurement were applied to the same CT images. We therefore performed a two tailed paired *t*‐test with a confidence interval of 95% to compare V_100%_, D_0.3cc_‐Cord, and D_1cc_‐Bowel of institution 1 Cyberknife to the other platforms and assess for target dose conformity. We used Bonferroni correction to account for multiple comparisons. D_1cc_‐Bowel and D_0.3cc_‐Cord in each institution were represented in value plots. All statistical analysis was done using Minitab^®^ version 17.3.1 (Minitab Inc., State College, PA).

## 3 RESULTS

**Figure 2:**
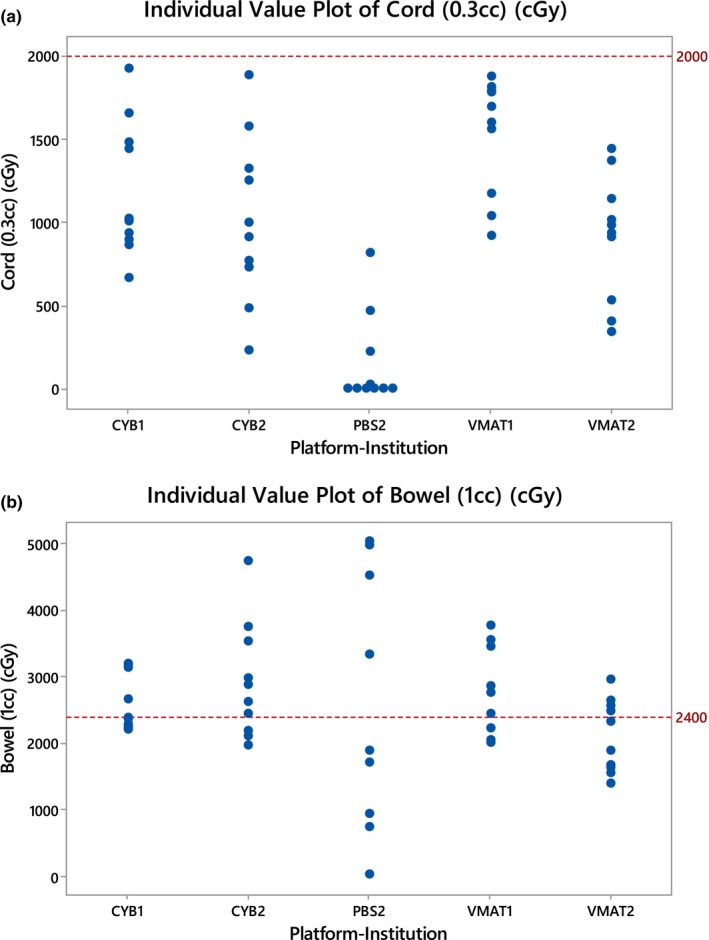
(a) Value Plot for D_0.3cc_‐Cord and (b) D_1cc_‐Bowel. CYB1: Cyberknife at institution 1, VMAT1: VMAT at institution 1, CYB2: Cyberknife at institution 2, VMAT2: VMAT at institution 2, PBS2: Proton Therapy at institution 2.

Table 2 shows the mean and the SD of each of all seven variables. Tumor coverage was excellent while also sparing the ipsilateral kidney. The V_100%_ was greater than or equal to 97.4% for all the platforms and the V_14Gy_ ranged between 45.6% (VMAT — Institution 1) and 65.1% (Cyberknife — Institution 2). Mean V_100%_ was the lowest for Cyberknife at institution 1. The D_0.3cc_‐Cord constraint was satisfied for the five platforms [Fig. 2(a) and Table 2]. For several cases, D_1cc_‐Bowel constraint was not achieved [Fig. 2(b)]. The mean D_1cc_‐Bowel satisfied the dose constraint only for VMAT — Institution 2, while the other platforms had slightly higher means ranging between 1.76 Gy (Cyberknife — Institution 1) to 5.14 Gy (Cyberknife — Institution 2) above the dose constraint (Table 2). Table 3 show the *P*‐values and the 95% confidence intervals of the paired *t*‐test for V_100%_, D_0.3cc_‐Cord, and D_1cc_‐Bowel. Using Bonferroni correction to account for multiple comparison, the *P*‐value for V_100%_ was statistically significant (*P* < 0.0125) in two out of four comparisons: Cyberknife — Institution 1 vs. VMAT — Institution 2 and Cyberknife — Institution 1 vs. PBS — Institution 2. For D_0.3cc_‐Cord, the *P*‐value was statistically significant (*P* < 0.0125) in three out of four comparisons: Cyberknife — Institution 1 vs. VMAT Institution 1 and 2 and Cyberknife — Institution 1 vs. PBS — Institution 2. The V_14Gy_ values are symmetric across the five platforms, ranging between 20% and 100% except for one value. The constraints were satisfied across all five platforms for D_5%_‐Contralateral K, D_1cc_‐Stomach, and V_17Gy_‐Liver.

**Table 2:** Dosimetric variables

Mean (min–max)	Institution 1		Institution 2		
CTV (cc)	80.12 (20.8–328.8)		81.68 (19.61–318.48)		
Mean (SD)	Cyberknife	VMAT	Cyberknife	VMAT	PBS
V100_%_ (%)	97.4 (1.35)	98.8 (1.52)	97.8 (2.26)	99.0 (0.65)	99.7 (0.20)
V14 Gy (%)	65.1 (26.58)	60.9 (24.25)	55.2 (18.94)	45.6 (18.18)	62.3 (21.50)
D1 cc‐Bowel	2576.2 (421.21)	2724.5 (653.71)	2913.8 (871.00)	2101.9 (548.61)	2806.2 (1973.29)
D1 cc‐Stomach	1699.7 (601.31)	1131.2 (841.34)	1352.91 (832.23)	802.89 (987.09)	696.99 (1611.75)
D0.3 cc‐Cord	1186.3 (406.45)	1522.6 (349.83)	1013.1 (500.84)	903.9 (377.25)	152.0 (278.02)
D5_%_‐Contralateral K	262.1 (153.17)	878.1 (359.68)	314.0 (407.93)	246.3 (100.57)	0 (0)
V17 Gy‐Liver (%)	11.3 (15.09)	4.1 (6.50)	9.0 (7.55)	10.5 (11.20)	6.8 (4.50)

**Table 3:** Paired *t*‐test for V_100%_, D_0.3cc_‐Cord, and D_1cc_‐Bowel

Paired *t*‐test: *P*‐value (95% CI* for mean difference)	Institution 1	Institution 2		
Institution 1 Cyberknife	VMAT	Cyberknife	VMAT	PBS
V_100%_	0.071 (−0.142, 2.882)	0.738 (−1.821, 2.477)	0.011 (0.442, 2.664)	0.01 (1.287, 3.259)
D_0.3cc_‐Cord	0.008 (−111.5, 561.1)	0.224 (−473, 127)	0.008 (−470.2, −94.6)	0.00 (−1275, −794)
D_1cc_‐Bowel	0.563 (−411, 707)	0.277 (−323, 998)	0.047 (−940, −9)	0.739 (−1286, 1746)

## 4 DISCUSSION

SBRT is currently under investigation as an alternative treatment for high risk surgical patients with RCC. In fact, ablative radio‐surgery might be the only curative choice for a subset of patients who are also not candidates for tyrosine‐kinase inhibitors. In our initial Phase I trial, robotic radiosurgery using CyberKnife was the platform of choice due to the ability to track tumor motion with respiration using fiducial gating. This study highlights the feasibility of offering SBRT for patients using other platforms in centers where robotic surgery is unavailable.

Compared to Cyberknife, other platforms provided equivalent (VMAT Institution 1 and Cyberknife Institution 2) or superior coverage (VMAT and PBS Institution 2) to the target volume as measured by the V_100%_. Differences in dose distribution among different platforms reflects the different characteristics of treatment delivery technique rather than inter‐operator variations in contouring. However, this is expected to lead to similar clinical outcomes since local tumor control saturates beyond a certain biologically effective dose usually in the range of 30–45 Gy delivered with SBRT fractionation schedules.^8^

The potential OAR toxicity associated with SBRT remains a limiting factor for adequate dose delivery. While VMAT in institution 1 was associated with higher D_0.3cc_‐Cord, VMAT and PBS planning at institution two resulted in lower D_0.3cc_‐Cord. With the OAR constraints used in our Phase I trial and for this study, no grade 3 or 4 toxicities were reported.^7^ These constraints were developed initially for the institution 1 protocol prior to the initiation of the phase I trial. Since then, the International Radiosurgery Oncology Consortium for Kidney (IROCK) consortium has been adopted internationally to help guide uniform dose constraints.^9^ These constraints are likely conservative and were strictly met for all the organs except the D_1cc_‐Bowel. In this feasibility study, no specific constraint optimization algorithm was used and the PTV expansion can be relaxed in cases were bowel toxicity is a concern. Regardless, the D_1cc_‐bowel should not be regarded as an absolute contraindication for RCC SBRT and treatment decisions should rather be based on a risk‐to‐benefit ratio in patients with no other alternative treatment options for a life‐threatening malignancy. Even with more permissive small bowel doses, the risk of grade 3 or worse toxicity remains low and is only around 10%.^10^

Two major limitations of this study are the lack of 4D motion studies and the difference in planning techniques among institutions. In general, abdominal organ respiratory motion is in the supero‐inferior direction with less than 2 mm displacement in the antero‐posterior and lateral directions.^11^ Motion studies account for intra‐fraction respiratory motion of the kidney. While passive motion‐management techniques such as internal target volume (ITV) are often used, the active techniques such as gating and tracking show better overall dose sparing.^12^ In the phase I protocol, image acquisition, target localization, and alignment correction were repeated during treatment delivery at intervals of 30–60 s.^7^ To limit respiratory motion, 4D‐CT images can be acquired with abdominal compression and plans could therefore incorporate an ITV. Contrary to gated radiotherapy, using an ITV would lead to an increase in the volume of the normal tissue irradiated. Given the large kidney size (3 × 6 × 12 cm^3^), the relatively small respiratory‐induced motion may mitigate the risk of non‐gated treatment delivery,^13^ especially that near complete sparing of a substantial volume of the kidney is usually associated with compensatory preservation of the renal function.^14^ Compared to Cyberknife, VMAT and PBS have the advantage of decreased treatment times,^15^ which decreases the risk of intra‐fraction movement, increases the number of patients treated per day, and provides more comfort to the patient.^15^ Finally, difference in planning techniques between institutions could have been a confounder and thus this dosimetric study should be interpreted as an initial proof of concept that needs further support in multi‐institutional settings.

## 5 CONCLUSION

This study suggests an excellent level of dosimetric consistency across different SBRT platforms at two different institutions. Future dosimetric studies can be improved by accounting for respiratory motion and integrating constraints from American Association of Physicists in Medicine (AAPM) Task Group 101^16^ and IROCK. The current results shows the dosimetric feasibility of the implementation of large multi‐center trials delivering renal SBRT using a myriad of platforms for RCC patients with high surgical risk. Further platform specific clinical studies using a unified planning technique are needed to address the advantage of each platform in renal SBRT and generate, accordingly, platform specific indications for SBRT in RCC.

## CONFLICT OF INTEREST

None.
